# Shall We Ask Congress for a Special Law Regulating Medical and Dental Patents?

**Published:** 1897-04

**Authors:** 


ARTICLE V.

### SHALL WE ASK CONGRESS FOR A SPECIAL LAW REGULATING MEDICAL AND DENTAL PATENTS?

Dental ethics and dental patents have been so inextricably mixed, that it is time that some effort should be made to straighten out the snarl. At present there are three distinct sets of opinions as to the righteousness of taking patents. Some men hold that no patents should be asked for by a man claiming to be professional. Others believe that they should not be considered unprofessional, merely because, having invented some useful appliance, they ask for governmental protection. A third-class cares nothing for the professional aspect, but insists upon his legal right to take patents. Curiously enough, there are many and good arguments offered by all three, though it is probable that the second claim is the one which should receive the support of the profession at large, provided that the present laws could be amended, in accordance with suggestions which will be made later. First, let us consider the subject analytically.

Whenever the real professional man asks the advocates of patents, "Why do you take a patent?" the invariable reply is "Why do you take a copyright " There is both similarity and difference between copyright and patent. They are dissimilar because, whereas the patent protects the invention, in its principle or essence, the copyright guards only the outward form. The holder of a patent may say: "That is my idea; you shall make no use of it without compensation to me." The professor of a copyright, conversely, cannot say: "Those are my ideas; you shall not use them without compensation to me." All that he can claim is: "That is my book; you shall not print those ideas, in that identical language, without my permission."

A man may write a work upon "Methods of Filling Teeth," for example. No other person may print the same ideas, in exactly the same language, nor under the same title. But all persons are privileged to write books upon the same subject, teaching identically the same methods, provided the language be different, and the title, "The Filling of Human Teeth," "The Filling of Teeth," "New Methods of Filling Teeth," Correct Methods of Filling Teeth," or any one of a hundred phrases, expressing the same idea, but utilizing a different sequence of words from those in the protected title.

Text books are the foundation stones ot knowledge. Without them; science would die, because the teachers might die without having been able to transmit their knowledge in a concrete form to any one of hundreds of their students; whereas, printed books stand as accurate records for all time. Professional men, as a rule, receive no reward, as a result of the possession of a copyright, which in any manner amply repays them for time devoted to the acquirement of that knowledge which they record in books. Nor is the copyright taken with a hope of reward, though reward should be ungrudgingly granted. The fact is that the production of a book of science is expensive, while the return is often chimerical. Consequently no publisher could be persuaded to undertake the production of a text book which had not been covered by coyyright. The publisher not being bound by any rules of dental ethics, and being the sole arbiter of what he will print, it is manifest that the copyright is a compulsory condition to publication. Without copyright there would be no text bo ks; without text books science would perish.

As to whether it should be considered professional or otherwise to take patents is worthy of some consideration. As being in a sense the act of a tradesman, and therefore the antithesis of professional spirit, it has been held, especially in the medical world, to be unprofessional. But in this age of progress, it is time that musty old doctrines should be revised to meet the modern advance in thought. Sentiment should have less force, and reason should hold sway. This does not mean that ethics should be set aside; but ethics, like all science, for ethics is a part of social science, is or should be subject of discussion, and if need be, of alteration.

In the last century it may have been true that the holding of a patent prevented a professional man from giving that attention to his duties which his patients had a right to exact. But to-day medical science has made great strides, and a tremendous proportion of all human diseases are cured by surgical interference. Surgery is now absolutely dependent upon mechanics, for without instruments, surgery is rendered powerless. Instruments can only be made in perfect form by manufacturers who devote capital to their production. Here then, as with the text book, we find that the foundation stones of surgery, its instruments depend upon the manufacturer, and no manufacturer will invest his money in their production without the protection which a government patent affords; this is not a theory; it is a fact.

The few "useful inventions" which have been "presented to the profession" have proven to be useless, because no manufacturer undertook their production. Several years ago a furnace for porcelain crown and bridge work was introduced and presented to the profession. Those present at the meeting, where this generosity was manifested, passed unanimously a resolution of thanks. Thus the inventor received the "thanks" of two or three hundred men, and the profession have never since had an opportunity to buy one of those furnaces. Why? There being no patent, no one manufacturer cared to invest the capital needed to make and advertise the article, creating a demand, which at the end could be enjoyed by other manufacturers, who would invest only after the demand had been shown to be existent.

It is, therefore, a short-sighted policy and opposed to the welfare of the professional body, to cast ethical reflections upon the medical or dental inventor who takes a patent upon an article of manufacture, which, when produced, is offered to all alike at a uniform price. Whether or not the inventor receives remuneration in cash fee, or royalty, is a matter aside from the question, and of no concern to the body of practitioners.

But there is another class of patents, sometimes taken by dentists, but as often by men who merely seize the opportunity to prey upon professional men. These are at present legal, though unrighteous; and being unrighteous, they should as soon as possible be declared illegal.

It has been shown that both copyright and patent are legitimate when used in the protection of, and therefore in the encouragement of the manufacture of, a uesful article. But when one man discovers, or imagines that he discovers a new method of procedure in practice, it should not be legal for him to exact tribute from all who use that method.

This may be considered from two aspects, the commercial and the ethical. From the commercial standpoint the ordinary inventor is permitted to exact and to receive a royalty, but in each instance he gives something tangible in exchange for the sum of money received; he furnishes a product of his own labor, or of labor hired with his own capital. He has money invested in his enterprise; he prepares and sells his patented article; there is a fixed price at which all may buy; and the patent protects him in the business which is founded upon his own ingenuity, and carried into effect with his own capital, it being immaterial to the argument that he may have associated with himself a capitalist in order to establish his business.

With a man who claims protection for a process it is different. He furnishes to the practitioner merely a set of directions. The thing produced, whether it be a crown, a bridge, a filling, a plate or any other dental appliance, is the product of the labor of the dentist. The dentist not only is expected to carry out the directions, the successful application of which depends upon his own skill, but it not infrequently will occur that unique peculiarities of a case, will require of him skillful modification of the patented method, in order to properly serve his patient. It might even be that such modification is so extreme that in the specified case a really new method has been devised. Nevertheless, under the present laws, it is legal for the holder of a patent upon a process, to exact a license, or a royalty, the latter being manifestly unjust in such a case as has been cited.

From the ethical standpoint we see, with but little analysis, that when dead scientists have freely bestowed the results of life-long study, thus furnishing the ladder upon which the living discoverer mounts to that point from which he "discovers" (?) his process, it is manifestly unjust that this one man, by governmental connivance, should be permitted to reap the entire reward. An example often points an argument, and a good one is at hand. There is in existence a patient, upon a process of producing anæsthesia. The discoveries of anæsthesia gave their secret to the world in the interest of suffering humanity. Had they not revealed the secret that such a thing as anæsthesia exists, how could this latter day scientist have "discovered" the best process of producing anæsthesia? How could he have invented a means of accomplishing that of which he had no knowledge?

The present law allows the patentee of a process to exact a license from all who practice the method; this also permits him to prevent all others from using the method. Is this just? Let us see.

The government sets up a standard which must be reached by all who wish to practice medicine or dentistry. To attain this the applicant must give several years of his life to study. Having done so, he is granted a license to practice. To practice what? The license should permit him to use the best known methods of cure in treating disease. Then why should the government give another individual the power to say to all practitioners: "I own the best method of treating that disease; if you wish to use it, pay me a license." This is not only a hardship against the men who have been required by the government to pay so dear a price for the license to practice, but in effect it is a fraud upon the community. The public having faith in the government, goes to a licensed practitioner, expecting the latest and best treatment; and he fails to receive it because of the misguided interference of the government, in granting to one man the monopoly of a method of cure.

The Dental Protective Association has done a holy work in fighting patents, which have been irksome to the dentists, and therefore burdensome to the people at large. But there never should have been any need for such co-operation of dentists, nor for the expenditure of the money which it has required. The profession should now unite in a request to Congress for the passage of a law, prohibiting the granting of any patent, upon any method or process of treating or curing human diseases or ailments. Is there precedent for such an act?

The patent laws of this country at present deny a patent for any contrivance aimed exclusively at the destruction of human life. In a medical sense this has special significance in that no patent can issue upon any method of preventing preventing pregnancy, or of producing abortion. If the government already thus widely restricts the patent from protecting those who *avowedly* aim their inventions or discoveries at the life of a human being, it is but a natural and proper sequence to refuse government protection to those who *covertly* aim at human life, by desiring the use of a cure known to them, except upon the payment of license or royalty.

In summing up these arguments let us admit the right of professional men to take copyright; let us encourage the brighter minds to invent instruments and appliances, and to cover them with patents; but let us ask our government to guard us in future from those human harpies who do not hesitate to prey upon the suffering of their fellows, who are willing to sit in idle luxury which they purchase with the tribute extorted from the pangs of human pain.—EDITORIAL in *Items of Interest.*

